# Measurement of heating coil temperature for e-cigarettes with a “top-coil” clearomizer

**DOI:** 10.1371/journal.pone.0195925

**Published:** 2018-04-19

**Authors:** Wenhao Chen, Ping Wang, Kazuhide Ito, Jeff Fowles, Dennis Shusterman, Peter A. Jaques, Kazukiyo Kumagai

**Affiliations:** 1 Indoor Air Quality Program, Environmental Health Laboratory, California Department of Public Health, Richmond, CA, United States of America; 2 Interdisciplinary Graduate School of Engineering Sciences, Kyushu University, Kasuga, Fukuoka, Japan; 3 Exposure Assessment Section, Environmental Health Investigation Branch, California Department of Public Health, Richmond, CA, United States of America; 4 Division of Occupational and Environmental Medicine, School of Medicine, University of California San Francisco, San Francisco, CA, United States of America; Beijing University of Posts and Telecommunications, CHINA

## Abstract

**Objectives:**

To determine the effect of applied power settings, coil wetness conditions, and e-liquid compositions on the coil heating temperature for e-cigarettes with a “top-coil” clearomizer, and to make associations of coil conditions with emission of toxic carbonyl compounds by combining results herein with the literature.

**Methods:**

The coil temperature of a second generation e-cigarette was measured at various applied power levels, coil conditions, and e-liquid compositions, including (1) measurements by thermocouple at three e-liquid fill levels (dry, wet-through-wick, and full-wet), three coil resistances (low, standard, and high), and four voltage settings (3–6 V) for multiple coils using propylene glycol (PG) as a test liquid; (2) measurements by thermocouple at additional degrees of coil wetness for a high resistance coil using PG; and (3) measurements by both thermocouple and infrared (IR) camera for high resistance coils using PG alone and a 1:1 (wt/wt) mixture of PG and glycerol (PG/GL).

**Results:**

For single point thermocouple measurements with PG, coil temperatures ranged from 322 ‒ 1008°C, 145 ‒ 334°C, and 110 ‒ 185°C under dry, wet-through-wick, and full-wet conditions, respectively, for the total of 13 replaceable coil heads. For conditions measured with both a thermocouple and an IR camera, all thermocouple measurements were between the minimum and maximum across-coil IR camera measurements and equal to 74% ‒ 115% of the across-coil mean, depending on test conditions. The IR camera showed details of the non-uniform temperature distribution across heating coils. The large temperature variations under wet-through-wick conditions may explain the large variations in formaldehyde formation rate reported in the literature for such “top-coil” clearomizers.

**Conclusions:**

This study established a simple and straight-forward protocol to systematically measure e-cigarette coil heating temperature under dry, wet-through-wick, and full-wet conditions. In addition to applied power, the composition of e-liquid, and the devices’ ability to efficiently deliver e-liquid to the heating coil are important product design factors effecting coil operating temperature. Precautionary temperature checks on e-cigarettes under manufacturer-recommended normal use conditions may help to reduce the health risks from exposure to toxic carbonyl emissions associated with coil overheating.

## Introduction

Use of electronic cigarettes (e-cigarettes), also referred to as “vaping”, has grown rapidly in popularity, especially among youth [1]. E-liquids generally contain propylene glycol (PG) or glycerol (GL) (or a mixture of both) as a nicotine solvent, along with or without nicotine, water, and flavorants. E-cigarette devices usually consist of the same basic components, including a battery, an atomizer (heating coil), and an e-liquid container or cartridge, or sometimes a combined atomizer and cartridge called a “clearomizer” or “cartomizer.” However, design feature details vary widely among the many hundreds of commercially available e-cigarette products and their replaceable components [2, 3]. Unfortunately, there is a general lack of product quality control, documentation of design parameter specifications, and instructions for use [4]. In 2016, the U.S. Food and Drug Administration (FDA) finalized a rule extending their regulatory authority to all Electronic Nicotine Delivery Systems (ENDS), including e-cigarettes. However, the specific product design features/parameters to be regulated have not yet been determined. In order to support effective policies, it is important to closely examine the features of different product designs and their potential impact on toxic chemical emissions.

Coil heating temperature is a key design and operating parameter affecting the amount and composition of aerosol emitted from e-cigarettes, as well as the sensorial quality perceived by e-cigarette users [5–7]. One concern related to the heating temperature, especially overheating, is the elevated exposure to toxic carbonyl compounds (formaldehyde, acetaldehyde, acrolein, etc.) due to the thermal decomposition of e-liquids around a heated coil during vaping [6–8]. This may be a particularly important concern for e-cigarettes that use a “top-coil” clearomizer, because its heating coil is placed close to the mouthpiece and it commonly uses long wicks to deliver e-liquid to the coil. Recent studies have revealed high emissions of toxic carbonyls with this design (See S1 Fig and S1 Table in Supporting Information for a summary of formaldehyde emission rate vs. applied power level) [9–13]. These studies used a 1:1 mixture of propylene glycol and glycerol (PG/GL), with or without nicotine and water, as the test e-liquid, and used an adequate liquid fill level. Results between these studies were inconsistent, with approximately five orders of magnitude difference in formaldehyde formation, from below the detection limit to as high as 97 μg/puff. These results have caused a wide debate on the conditions of safe use for this product design. It is generally acknowledged that a higher voltage or power level would lead to higher coil temperature and therefore higher carbonyl formation rates. However, a very high formaldehyde formation rate was observed even at the lowest applied power in one study [13]. Because coil temperatures were not reported in these studies, further research is needed to identify causative factors affecting heating coil temperature, and to determine the relationship of applied power and heating temperature under various coil conditions.

Many factors, such as coil material and resistance, battery voltage, atomizer/wick design, e-liquid composition and fill level, and vaping topography (puffing time, interval, or volume) may affect the operating temperature of e-cigarettes. Only limited studies have been done on quantitative and direct temperature measurement at or near heating coils, reporting a wide range from 40 to 950°C depending on measurement location, measurement technique, test condition, and the type of e-cigarette used (See S2 Table in Supporting Information for details) [6, 14–19]. Two measurement techniques have been used in these studies. A thermocouple is an inexpensive and convenient method to conduct point measurements of temperature. For example, Zhao et al. [17] demonstrated that it is possible to insert a thermocouple probe into the cartridge of an e-cigarette from the end-hole and contact the heating coil (and/or wick), making it possible to measure the operating coil temperature while simultaneously sampling aerosol emissions. Although convenient, a thermocouple reading cannot provide information on the spatial temperature distribution across a heating coil at a given instant. An infrared (IR) camera (also called thermographic camera) has also been used, but often without puff flow so that the coil can be exposed to a camera. Currently, no standardized test protocol has been developed for coil temperature measurement.

The objective of this study was to determine the effect of applied power settings, coil wetness conditions, and e-liquid compositions on the coil heating temperature for e-cigarettes with a “top-coil” clearomizer, and to make associations of coil conditions with emission of toxic carbonyl compounds by combining results herein with the literature.

## Methods

### E-cigarette device and e-liquids

All experiments conducted in this study used a second generation e-cigarette device with a single “top-coil” clearomizer (EGO-CE6) that was purchased online in 2016. The EGO-CE6 had essentially the same coil/wick design as earlier versions of the EGO-CE product, but had a detachable and easy-to-change coil head, and a larger tank that could hold up to 2.4 mL of e-liquid. An upgrade to the EGO variable voltage battery was purchased together with a starter kit. The battery was rated at 1100 mAh with an adjustable voltage setting of 3.0 ‒ 6.0 V at 0.1V intervals. Virtually identical, replaceable coil heads were available for this device at different resistance levels, and three types of coil heads labelled low (LR), standard (SR), and high resistance (HR) were purchased. The coil resistances were measured in house using a multimeter (Fluke 87V True RMS Multimeter) and were determined to be 2.2 ± 0.2, 2.7 ± 0.2, and 3.7 ± 0.2 Ω for the LR, SR, and HR coils, respectively.

PG (> 99.5%, Sigma-Aldrich, USA) was used as the main test liquid to compare the effects of different coil wetness conditions and applied power settings on temperature. For a subset of coil settings, the effect of e-liquid composition was further investigated with PG and a 1:1 (wt/wt) mixture of PG/GL (GL: > 99.5%, Sigma-Aldrich, USA).

### Temperature measurement

**Fig 1:**
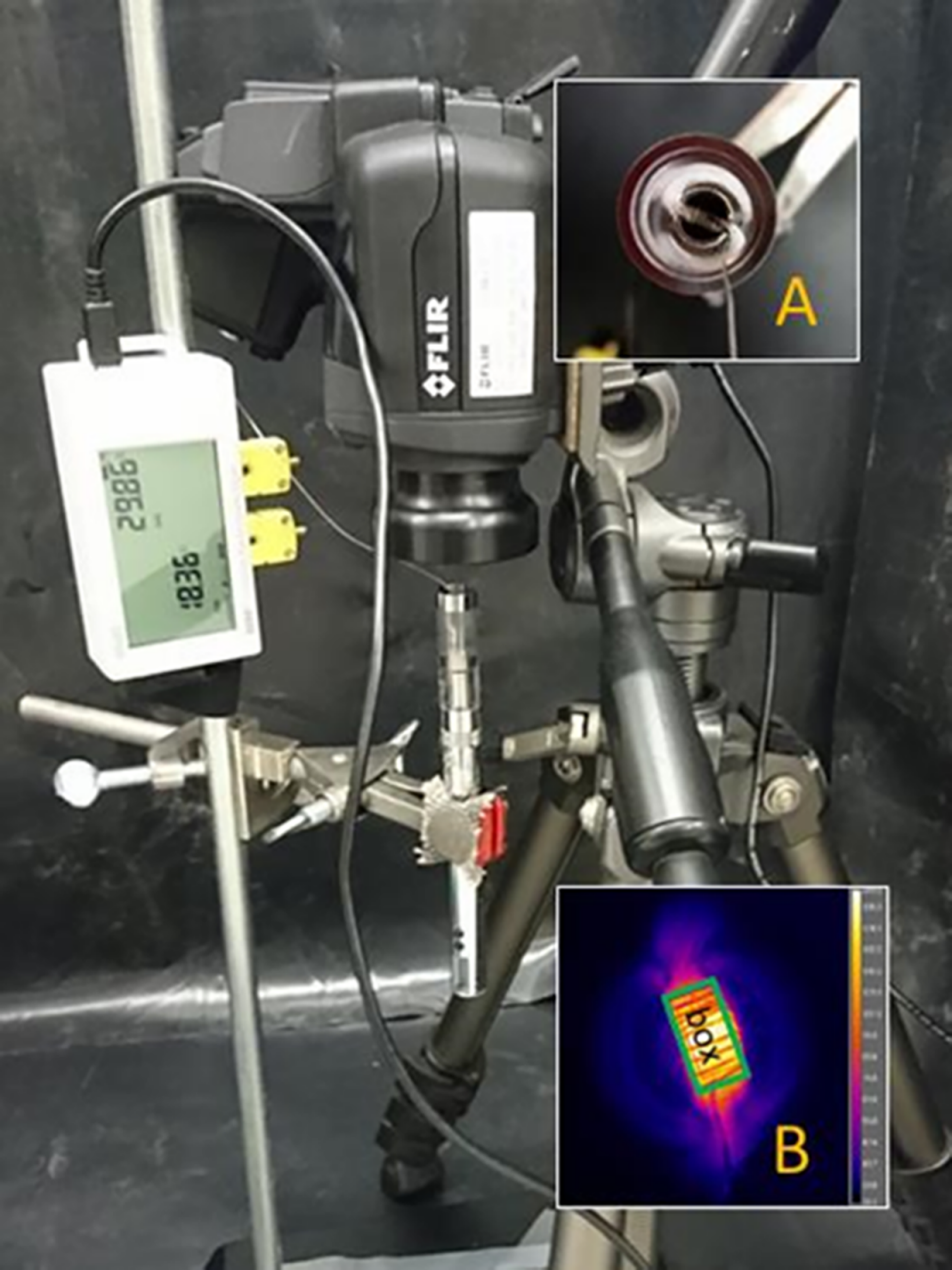
**Experimental setup for determination of coil heating temperature using a thermocouple and an infrared (IR) camera:** (A) typical position of the thermocouple on a coil head; and (B) details of the heating coil section (green box) from which minimum, mean, and maximum temperatures were determined using an IR camera.

A rapid response Type-K thermocouple of 0.25 mm diameter (OMEGA's Quick Disconnect Thermocouples, OMEGA, USA) was used in all experiments, while an IR camera (Model T440 with 2X 50 μm close-up lens, FLIR Systems, USA) was additionally used for a subset of coil conditions. The measurement tolerance and measurement range of the thermocouple probe were the greater of 1.1°C or 0.4%, and 0–1250°C, respectively. Thermocouple temperature was recorded using a data logger (UX120-014M, Onset, USA). The accuracy and resolution of the thermocouple data logger were ± 0.7°C ± thermocouple probe accuracy, and 0.04°C, respectively. The IR camera carried a manufacturer’s certification of calibration. For all experiments, its temperature range and emissivity index (ε) were set at 0–650°C and 0.96, respectively. To minimize the impact of reflected IR radiation from the surroundings, an opaque, black plastic sheet was used to form a small (~ 1m × 1m) surrounding “wall” surface (Fig 1). All tests were conducted in a laboratory environment with temperature maintained at 21± 3°C.

The impact of voltage setting, coil resistance, e-liquid fill level, degree of coil wetness, e-liquid composition, and measurement technique on coil temperature was examined as follows:

1. Different e-liquid fill levels in a clearomizer

PG was used as the test liquid. The e-cigarette device was set vertically to keep the length of both sides of the wick even in the e-liquid. The mouthpiece was removed to attach the thermocouple directly to the heating coil (wire) to more clearly observe the position of the contact point (see Fig 1A). Once the thermocouple position was fixed, measurements of the e-liquid fill conditions were conducted without touching the thermocouple:

1.1 Dry condition–Coil temperature was measured in the absence of any test liquid at voltage settings of 3, 4, 5, and 6 V. This condition represented the extreme worst scenario with an e-liquid fully exhausted.

1.2 Wet-through-wick condition–The clearomizer was filled to 1.6 mL without directly dripping liquid on the heating coil; after ≥30 minutes of wick wetting, coil temperature was measured as in 1.1. This condition represented the normal wick/coil wetness encountered in real vaping.

1.3 Full-wet condition–The clearomizer was filled to >2.3 mL, i.e., the entire coil was in direct and full contact with the liquid, and coil temperature was measured as in 1.1. This represented the ideal condition that fully excluded the possibility of a coil “drying up”. However, because the coil head was in the airflow pathway, e-liquid could (and did) easily leak through the bottom air holes.

The above measurements were conducted for low, standard, and high resistance coils respectively, each repeated with four coil heads (LR #1–4, SR #1–4, and HR #1–4). The same e-cigarette, fully recharged but with a new coil head, was used for each series of measurements. Temperature measurements were stopped at 5 V in some experiments (mostly for LR coils) due to a concern for coil failure at high voltage. At each voltage setting, the thermocouple readings were recorded every second over five consecutive button activation cycles, simulating puff cycles. For each cycle, the activation button was engaged for 4 seconds every 30 seconds, as recommended by Farsalinos et al [20]. The coil temperature was allowed to return to < 30°C between tests at different voltage settings.

2. Different levels of coil wetness

The following tests were conducted using a previously tested high resistance coil (HR #4) to determine the effect of additional levels of coil wetness:

2.1 Dry condition–see 1.1, above, repeated as a control condition.

2.2 Direct-dripping condition– 25 mg of PG was added to the coil using a syringe, and coil temperature was measured at 3 and 6 V, as above, to determine temperature change as a coil rapidly dried.

2.3 Liquid-drenched wick condition–Both sides of a wick were immersed in PG for 2 minutes after which the wick was exposed to room air, and coil temperature was measured as in 2.2 to determine temperature change when there is limited liquid supply from a wick.

3. Different e-liquid compositions

Further tests were conducted for high resistance coils only (HR #5–6) using PG and a 1:1 PG/GL mixture as test e-liquids. Thermocouple and IR camera measurements were made simultaneously for each coil under different e-liquid fill levels in the clearomizer (see protocol 1). The minimum, average, and maximum temperatures of a defined section of the heating coil on the IR camera image, i.e., the green box area shown in Fig 1B, were recorded.

Table 1 summarizes all temperature measurements conducted in this study.

**Table 1:** Summary of temperature measurement conditions.

Coil #	Coil Resistance	E-liquid	Different e-liquid filling levels?	Dry coil vs. direct dripping vs. liquid-drenched wick?	Thermocouple measurement?	IR Camera measurement?
HR1	High	PG	Y		Y	
HR2	High	PG	Y		Y	
HR3	High	PG	Y		Y	
HR4	High	PG	Y	Y	Y	
HR5	High	PG	Y		Y	Y
HR6	High	1:1 PG/GL	Y		Y	Y
SR1	Standard	PG	Y		Y	
SR2	Standard	PG	Y		Y	
SR3	Standard	PG	Y		Y	
SR4	Standard	PG	Y		Y	
LR1	Low	PG	Y		Y	
LR2	Low	PG	Y		Y	
LR3	Low	PG	Y		Y	
LR4	Low	PG	Y		Y	

LR = low resistance (2.2 ± 0.2 Ω), SR = standard resistance (2.7 ± 0.2 Ω), HR = high resistance (3.7 ± 0.2 Ω), PG = propylene glycol, GL = glycerol.

## Results and discussion

### Heating coil temperature measurement by thermocouple

#### Effect of e-liquid fill level and test repeatability

**Fig 2:**
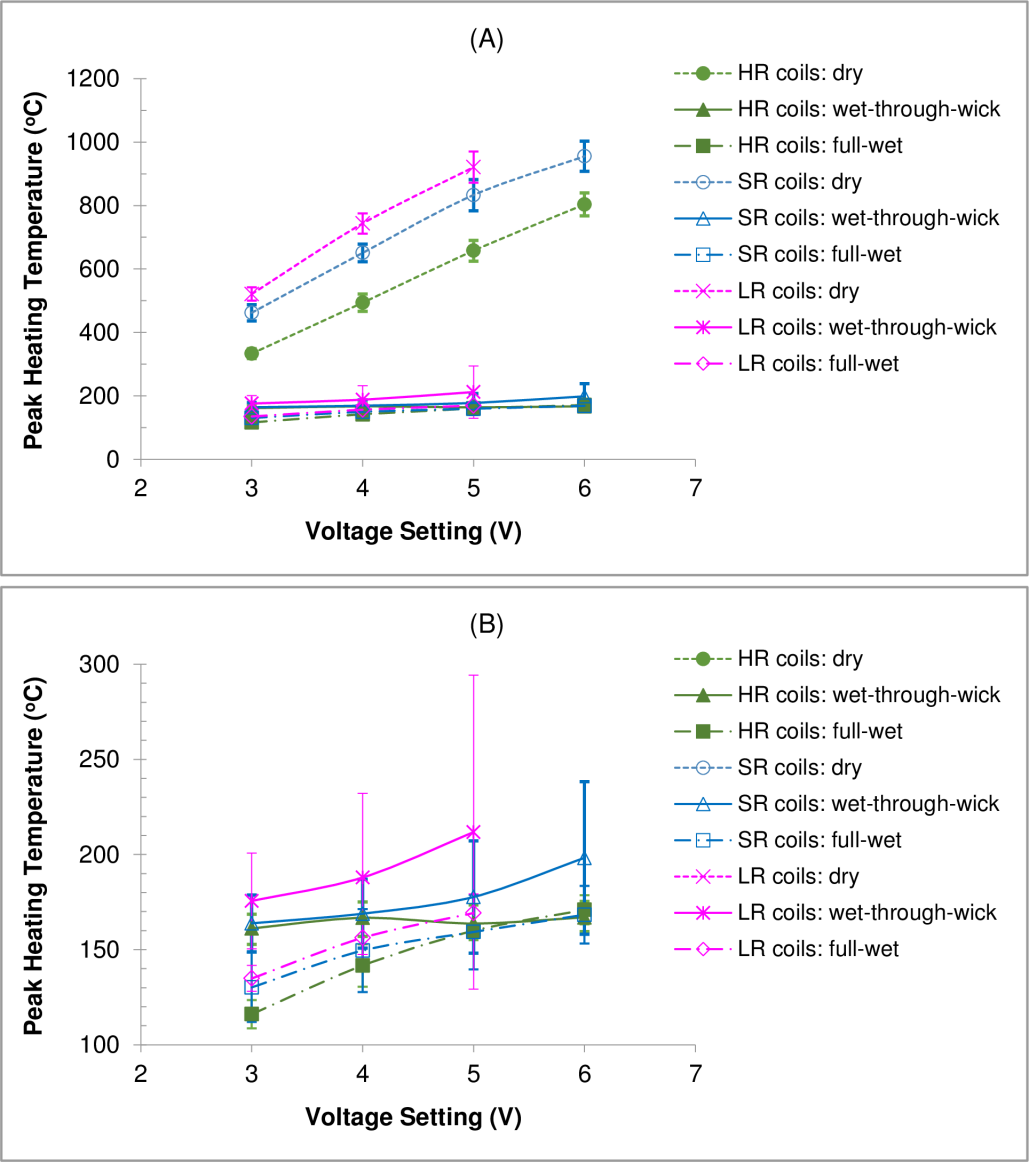
**Peak heating temperature for different coil resistances and e-liquid fill levels as a function of voltage setting: (A) full scale; and (B) enlarged scale.** Note: the average temperature ± one standard deviation is presented for four coils tested at each resistance level; LR = low resistance, SR = standard resistance, and HR = high resistance; button activation cycle = 4 s activation every 30 s; test liquid = propylene glycol.

**Fig 3:**
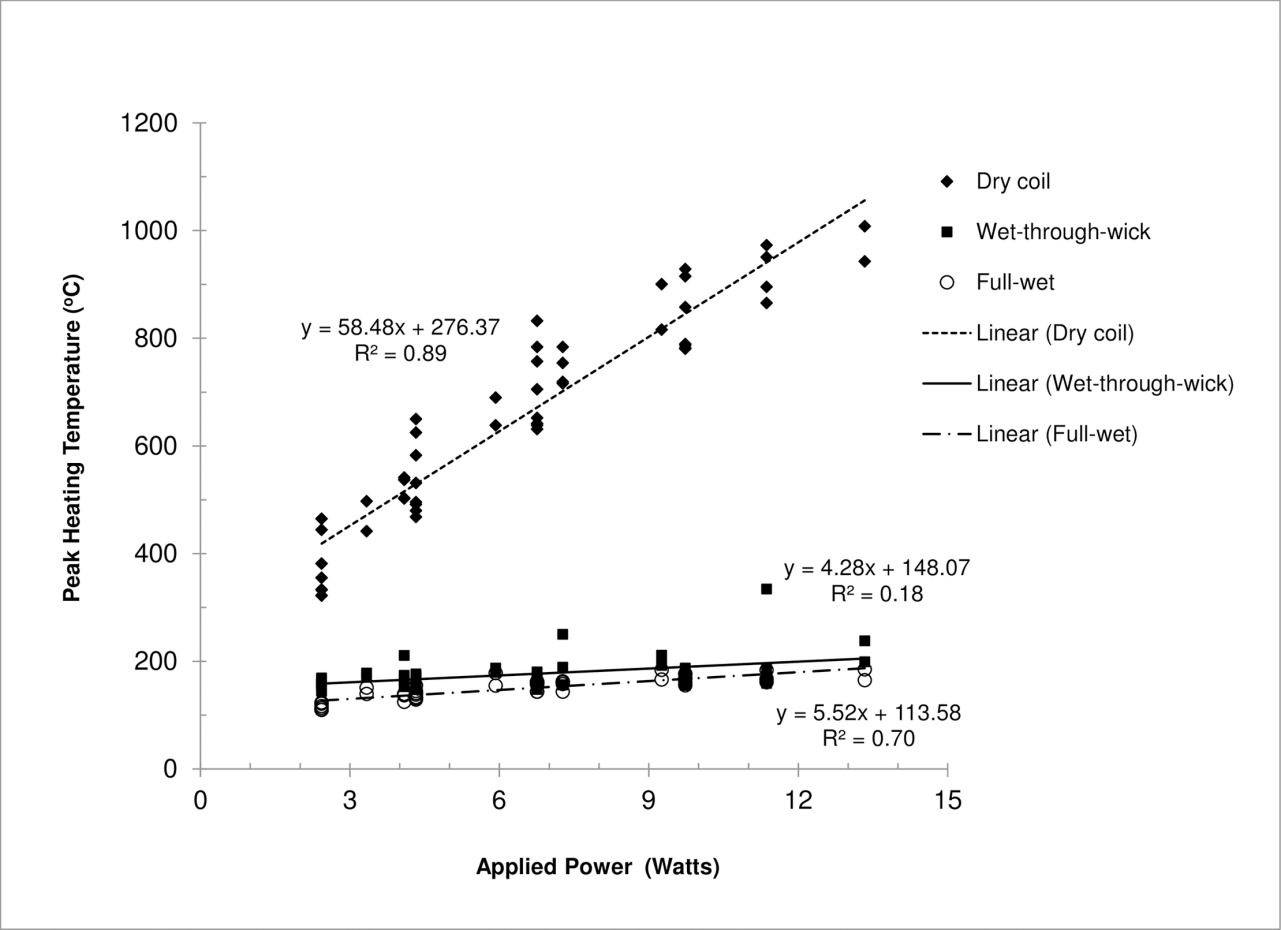
Peak heating temperature measured for different voltage settings, coil resistances, and e-liquid fill levels as a function of applied power. Note: button activation cycle = 4 s activation every 30 s; test liquid = propylene glycol.

Fig 2A and 2B show the mean peak temperatures measured under different e-liquid fill levels as a function of voltage setting for coils LR #1–4, SR #1–4, and HR #1–4. For each coil, the peak temperatures measured during the last four of five consecutive button activation cycles were averaged first. These temperatures were then used to determine the mean peak temperature and standard deviation shown in Fig 2A and 2B for the four coils tested at each resistance level (LR, SR, and HR). Fig 3 shows the peak coil temperature as a function of applied power for all—coils. The applied power was calculated based on the voltage and coil resistance according to Ohm’s law. Results for the three conditions were as follows:

- Under full-wet conditions, measured peak coil temperatures ranged from 110°C at the lowest power input of 2.4 W to 185°C at the highest power input of 13.3 W, which were all below the boiling point of PG (188°C). There was a positive correlation (R^2^ = 0.70) between peak heating temperature and applied power. Increase of applied power only slightly increased heating coil temperature.
- The wet-through-wick test represented a typical e-liquid fill condition for common use (2/3 of a 2.4-ml capacity). It is notable that the peak temperatures (145 to 334°C) were generally higher than those observed under full-wet conditions, suggesting that under common use, a coil may not always be fully wet. Additionally, we saw more variation among different coil heads under the wet-through-wick conditions. For example, temperature increase with increasing voltage was much more significant for LR #1 and SR #1 compared to others, resulting in larger standard deviations between these coils (Fig 2B). In common occurrence, such replacement coils/heads can be purchased from multiple online stores without detailed product specification, which may result in even larger performance variation.
- For dry coils, peak temperatures ranged from 322 to 1008°C. There was a stronger positive correlation (R^2^ = 0.89) between peak heating temperature and applied power compared to wet coil conditions. It is recognized that user awareness of a “dry puff” (i.e., a strong unpleasant taste due to liquid overheating) limits the occurrence of dry coil vaping [11]. However, if a wick cannot deliver sufficient e-liquid to the coil due to a manufacturing defect, it is possible that coil temperature will deviate significantly from normal use conditions towards much higher temperatures. Under such circumstances, the applied power can significantly affect coil temperature during coil drying.

#### Effect of coil wetness condition

**Fig 4:**
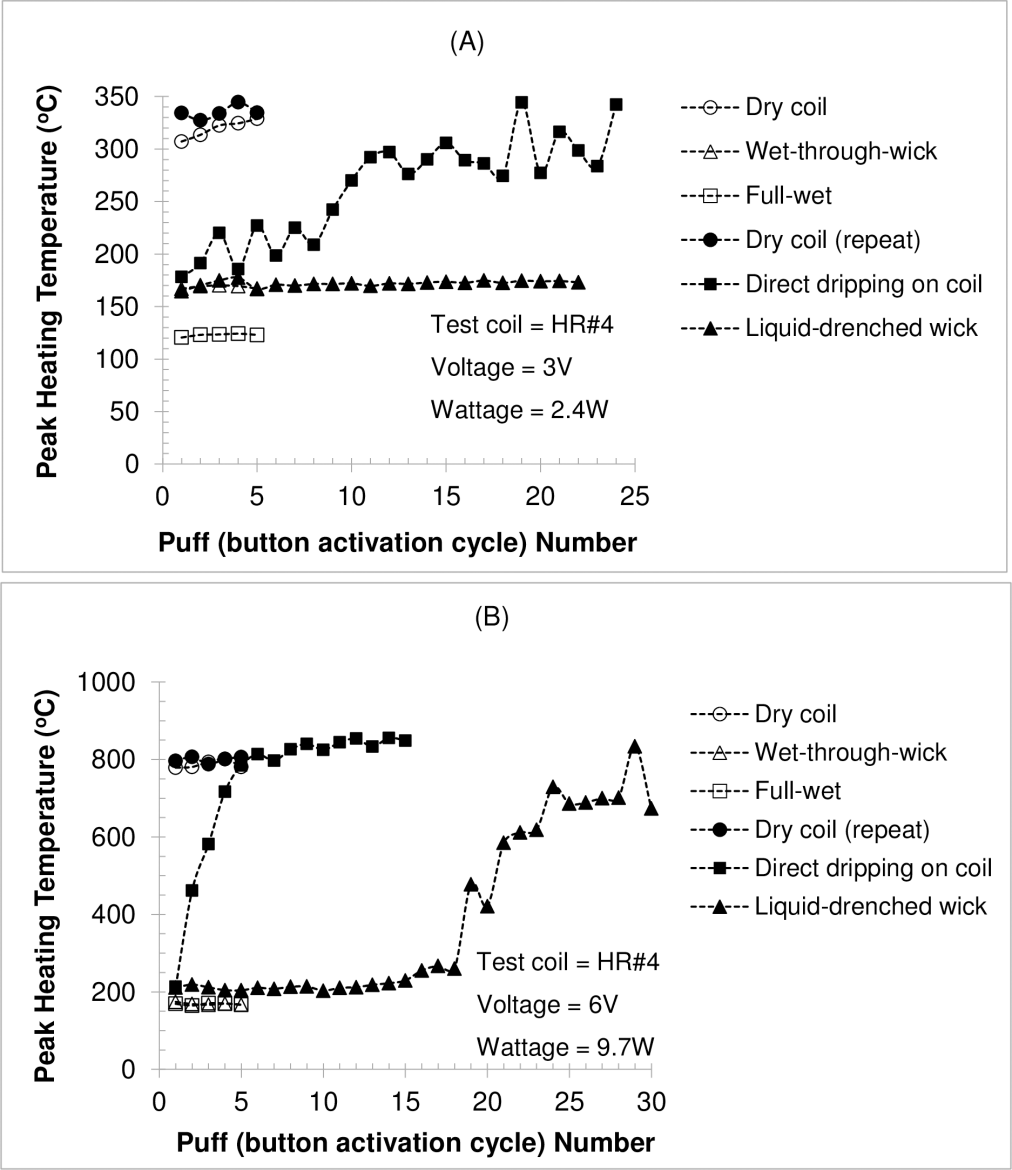
**Peak heating temperature measured for coil HR #4 under various coil conditions: (A) 3 V; and (B) 6 V.** Note: HR = high resistance; button activation cycle = 4 s activation every 30 s; test liquid = propylene glycol.

Fig 4A and 4B show peak heating temperatures of consecutive button activation cycles for HR #4 measured under various coil conditions (dry, direct-dripping, liquid-drenched wick, wet-through-wick, and full-wet) at 3 V and 6 V, respectively. As expected, peak coil temperatures during the first button activation cycle were similar under various wet coil conditions, but “direct-dripping” resulted in the quickest temperature increase towards a dry coil condition for consecutive cycles. Under this mode, the voltage/power level had the largest effect. Peak temperature reached that of a dry coil after 5 puffs at 6 V (or 9.7 W), while at 3 V (or 2.4 W) it reached only ~ 90% of dry coil temperature after 12 puffs. Talih et al. [16] measured the coil temperature of an e-cigarette with a direct drip atomizer (4.6 W battery output) and raised a concern about potentially higher temperatures due to “direct-dripping”. Their peak coil temperature was below 130°C during the first two puffs, then quickly increased to above 250°C during puff 3 and further to about 340°C during puff 4. Although our results confirm such a rapid temperature increase, the tested e-cigarette device was not meant to be used as a direct drip atomizer. For the liquid-drenched wicks, peak coil temperature was similar to the wet-through-wick condition during the entire test period at 3 V (Fig 4A). At 6 V, it was only slightly higher than the wet-through-wick condition at the beginning but increased rapidly and substantially towards the dry coil condition after puff 18 (Fig 4B). These data suggest that temperature measurement using e-liquid-drenched wicks may reasonably represent the actual wet-through-wick condition for the initial button activation cycles.

#### Effect of test e-liquid

**Fig 5:**
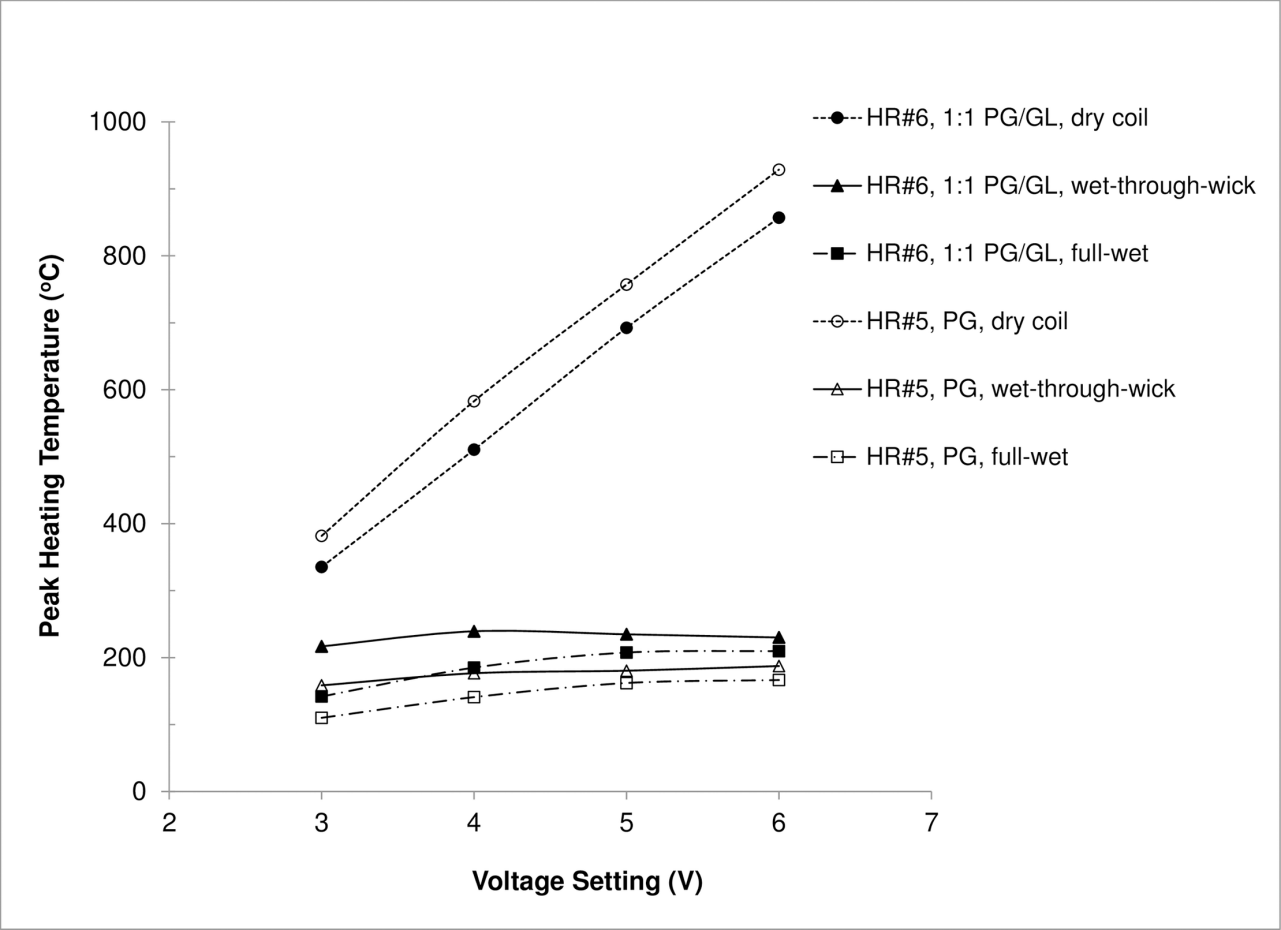
Peak heating temperature measured for different e-liquid fill levels and e-liquid compositions as a function of voltage settting. Note: HR = high resistance; button activation cycle = 4 s activation every 30 s; test liquid = propylene glycol (PG) or a 1:1 (wt/wt) mixture of propylene glycol and glycerol (PG/GL).

Fig 5 compares the peak HR coil temperatures under different e-liquid fill levels as a function of voltage setting for PG and a 1:1 (wt) PG/GL mixture. Despite the lower peak temperature of HR#6 compared to HR #5 under dry condition, which was probably due to coil performance variation and point measurement uncertainty, the peak heating temperature for 1:1 PG/GL (HR #6) was consistently higher than that for PG (HR #5) at all voltage settings under full-wet and wet-through-wick conditions. For example, under the wet-through-wick condition, the highest temperature observed was 188°C for PG and 244°C for PG/GL. These results indicate that e-liquid composition could significantly influence coil temperature, confirming a theoretical expectation [5, 21]. On the other hand, all the temperatures were still significantly lower than that of the dry coil, implying that the effectiveness of wick delivery is perhaps the most important design feature determining e-cigarette operating temperature range.

### Comparison between thermocouple and infrared thermography measurements

**Fig 6:**
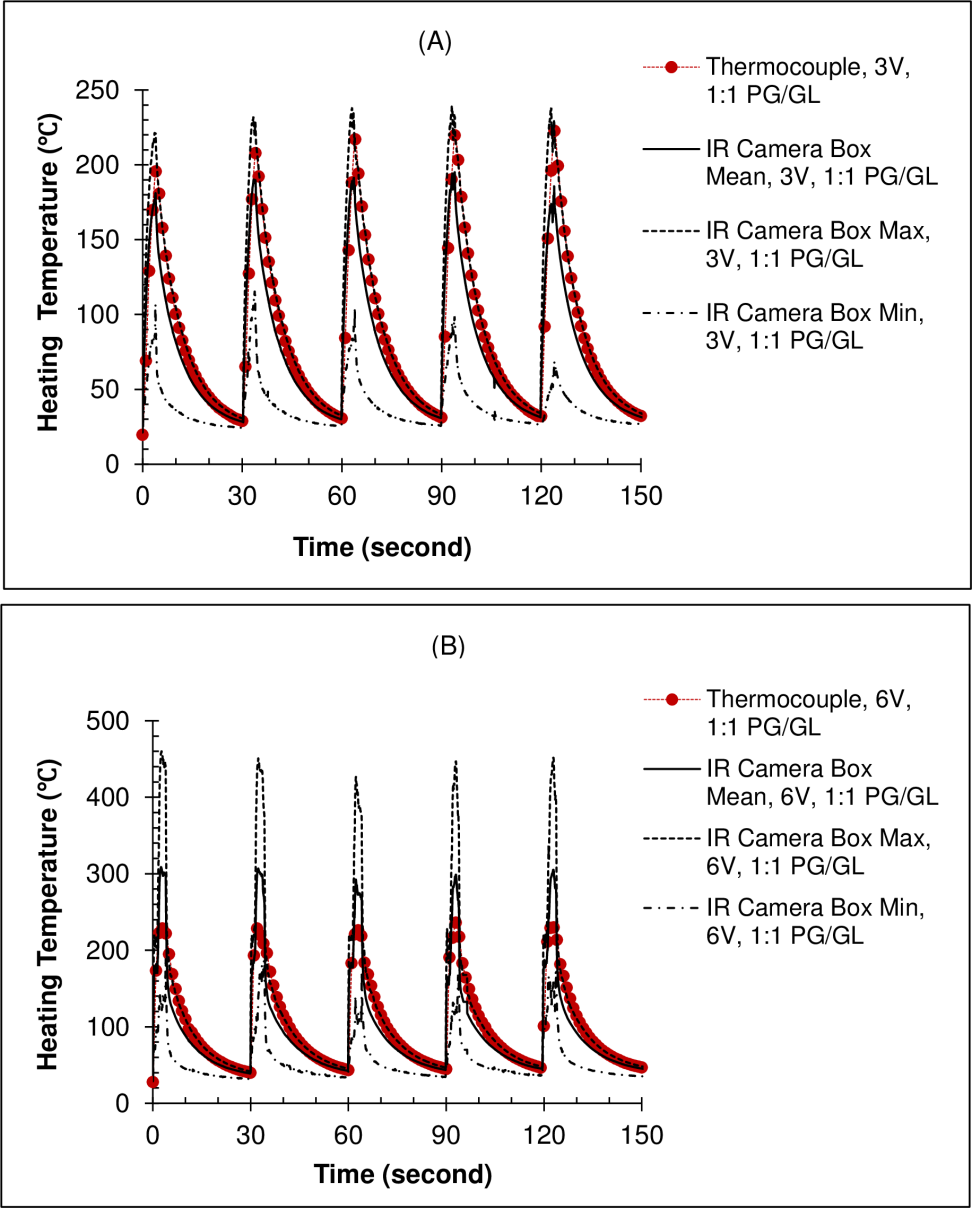
**Comparison of thermocouple and infrared (IR) camera temperature measurements for coil HR #6 under the wet-through-wick condition: (A) 3 V; and (B) 6 V.** Note: HR = high resistance; button activation cycle = 4 s activation every 30 s; test liquid = 1:1 (wt/wt) mixture of propylene glycol and glycerol (PG/GL); see Fig 1 for measurement set-up.

Table 2 summarizes temperature measurements by a thermocouple and an IR camera for the two wet coil conditions. Temperatures for dry coil conditions are not included because they exceeded the range-limit of the IR camera resulting in a plateau under high voltage settings. For tests using PG under full-wet condition, peak temperatures were abnormally high for the last cycle at 4 V and for the first cycle at 5 V in comparison to other button activation cycles. The reason for this occurrence was not clear; therefore, the temperatures of these two cycles are also excluded in Table 2. Examples of real-time measurements for the 1:1 PG/GL under wet-through-wick condition at 3 V and 6 V are shown in Fig 6A and 6B, respectively. There was no significant increase in the peak temperature over consecutive button activation cycles although the starting temperature increased slightly in subsequent cycles.

**Table 2:** Comparison of temperature measurements by thermocouple and infrared (IR) camera.

Coil condition	E-liquid	Voltage (V)	Coil Temperature (°C) ^a^				
			Thermocouple	IR Camera ^b^			
				Box Mean	Box Min	Box Max	Δ Box
wet-through-wick	PG	3	164	160	106	181	75
wet-through-wick	PG	4	180	169	128	188	60
wet-through-wick	PG	5	181	175	135	205	70
wet-through-wick	PG	6	188	247	172	381	208
full-wet	PG	3	112	146	100	172	71
full-wet	PG	4	134	166	127	185	58
full-wet	PG	5	164	168	130	186	56
full-wet	PG	6	169	171	133	202	69
wet-through-wick	PG/GL^c^	3	223	195	115	240	124
wet-through-wick	PG/GL	4	244	215	134	276	142
wet-through-wick	PG/GL	5	243	278	184	402	217
wet-through-wick	PG/GL	6	236	309	191	460	269
full-wet	PG/GL	3	148	172	112	209	97
full-wet	PG/GL	4	186	178	124	220	96
full-wet	PG/GL	5	218	189	131	235	104
full-wet	PG/GL	6	214	223	148	261	113

^a.^ Maximum peak temperature over five consecutive button activation cycles

^b.^Box Mean = mean temperature of the heating coil section (see green box defined in Fig 1B), Box Max = maximum temperature of the heating coil section, Box Min = minimum temperature of the heating coil section, Δ Box = Box Max–Box Min

^c.^PG/GL = 1:1 (wt) PG/GL

Compared to the thermocouple, the IR camera measurements show a more consistent and obvious trend of temperature increase with increasing voltage setting for all test conditions. The IR camera results also demonstrate that temperature was not uniform across a heating coil, especially under wet-through-wick and high voltage conditions. Zhao et al. [17] assumed that the temperature of the entire coil area was equal when using thermocouple measurements to study the effects of design parameters and puff topography on heating coil temperature. Our results indicate that this coil temperature uniformity assumption may not be true. Nevertheless, all thermocouple measurements were between the minimum and maximum temperatures of the heating coil section measured by the IR camera, and equal to 74% ‒ 115% of the coil mean, depending on test conditions. Both measurement techniques also revealed similar general trends, such as higher coil temperature for 1:1 PG/GL than PG alone under the same liquid fill level and voltage setting, and higher temperature under wet-through-wick than full-wet conditions for the same e-liquid and voltage setting. Therefore, a thermocouple may still be a useful and convenient tool to estimate e-cigarette operating temperatures.

### Associations among applied power, coil temperature, and formaldehyde formation

The IR camera measurements clearly showed higher temperature (e.g., up to 460°C for a 1:1 PG/GL mixture) as well as larger temperature non-uniformity across the heating coil at higher voltage/applied power (e.g., HR coil at 6 V or 9.7 W) under wet-through wick conditions. Our previous study under controlled heating temperatures using a tube reactor demonstrated a very rapid increase of carbonyl emissions as heating temperature increased above 270°C (e.g., from 0.96 ± 0.35 μg formaldehyde/ml-liquid at 270°C to 5.47 ± 0.72 μg formaldehyde/ml-liquid at 318°C for 1:1 PG/GL) [7]. This may explain why large formaldehyde emissions were always detected at higher voltage/applied power (e.g., ≥ 9 W). The results in published studies (see S1 Fig) differed substantially at lower voltage/applied power (e.g., 4–6.5 W). Our results suggest that high temperatures (e.g., up to 276°C for a HR coil at 4 V) were possible even at low voltage/applied power due to temperature non-uniformity and performance variation among different coil heads. Farsalinos et al. [11] stated that the issue of overheating and dry-puff conditions has been neglected in most laboratory studies evaluating e-cigarette aerosol emissions and that studying the dry puff phenomenon is important in e-cigarette research. However, since the “dry-puff” condition can only be identified by human subjects and there are individual differences in “dry-puff” detection, it is not possible to standardize a “dry-puff” condition. On the other hand, our study demonstrated that it is possible and straight-forward to measure coil temperature under certain standardized wet-through-wick or liquid-drenched wick conditions for button-activated devices. Measuring heating coil temperature under manufacturer-suggested e-liquid filling and voltage setting levels may be a good precautionary measure in order to minimize the possibility of “dry-puff” occurrence.

Additionally, it should be noted that even at an average temperature of 215°C, which was observed from our measurements for 1:1 PG/GL at relatively low applied powers and is within the reported range of operating temperatures for generating “pleasant” vapors [6], the formation rate of formaldehyde and thus the daily exposure of frequent e-cigarette users could still be significant and reach a level of concern for cancer risk [7].

### Limitations of the study

Only cyclic button activation was used to represent the puff cycle. The top of the clearomizer remained open and no puff flow was drawn through the e-cigarette device during temperature measurements. In real applications, puff airflow helps to carry away the heat generated at the coil and thus may lower peak temperature. However, the impact of airflow seems to be moderate. Zhao et al. [17] measured the heating coil temperature of four e-cigarettes under simulated use conditions and found that temperature decreased only ~ 10% when the puff flow rate increased from 0.5 to 2 L/min.

Our discussion of the associations among applied power, coil temperature, and formaldehyde formation was based on three separate pieces of information: coil temperature measurements, carbonyl formation under controlled heating temperatures, and the synthesis of data from prior studies that measured carbonyl formation using similar e-cigarettes. It would be more ideal and conclusive if one could simultaneously measure dynamic temperature change and carbonyl formation for such devices.

## Conclusions

This study established a simple and straight-forward protocol to systematically measure e-cigarette coil heating temperature under dry, wet-through-wick, and full-wet conditions. Although there were a few limitations, the study generated measurement-based evidence for temperature range possibly encountered under various coil conditions for e-cigarette with a “top-coil clearomizer” design. Results show that applied voltage or power was not the only factor determining coil operating temperature; wick design and ability to efficiently deliver e-liquid to the heating coil also played key roles. Higher power levels led to substantially higher coil temperatures under dry and sometimes wet-through-wick conditions, but there was much less temperature increase if the coil was always fully wet. Results also demonstrate that the composition of e-liquid could influence coil temperatures. Guidelines or regulations on e-cigarette device quality control and precautionary temperature checks under manufacturer-recommended normal use conditions may help to reduce the health risks from exposure to toxic carbonyl emissions associated with coil overheating.

## Supporting information

S1 Fig Formaldehyde emission rates in four studies of CE series or similar e-cigarette devices with single “top-coil” clearomizer design. Note: a 1:1 mixture of propylene glycol and glycerol (PG/GL), with or without nicotine and water, was used for tests summarized here.
(TIF)

S1 Table Details of e-cigarette settings and raw emission data for S1 Fig. (DOCX)

S2 Table Summary of temperature measurements at (or near) a heating coil reported in the literature. (DOCX)
