# Supplementary figures and images for: Measurement of heating coil temperature for e-cigarettes with a “top-coil” clearomizer
Source: PLoS One. 2018 Apr 19;13(4):e0195925. doi: 10.1371/journal.pone.0195925 (PMC5908153; doi:10.1371/journal.pone.0195925)

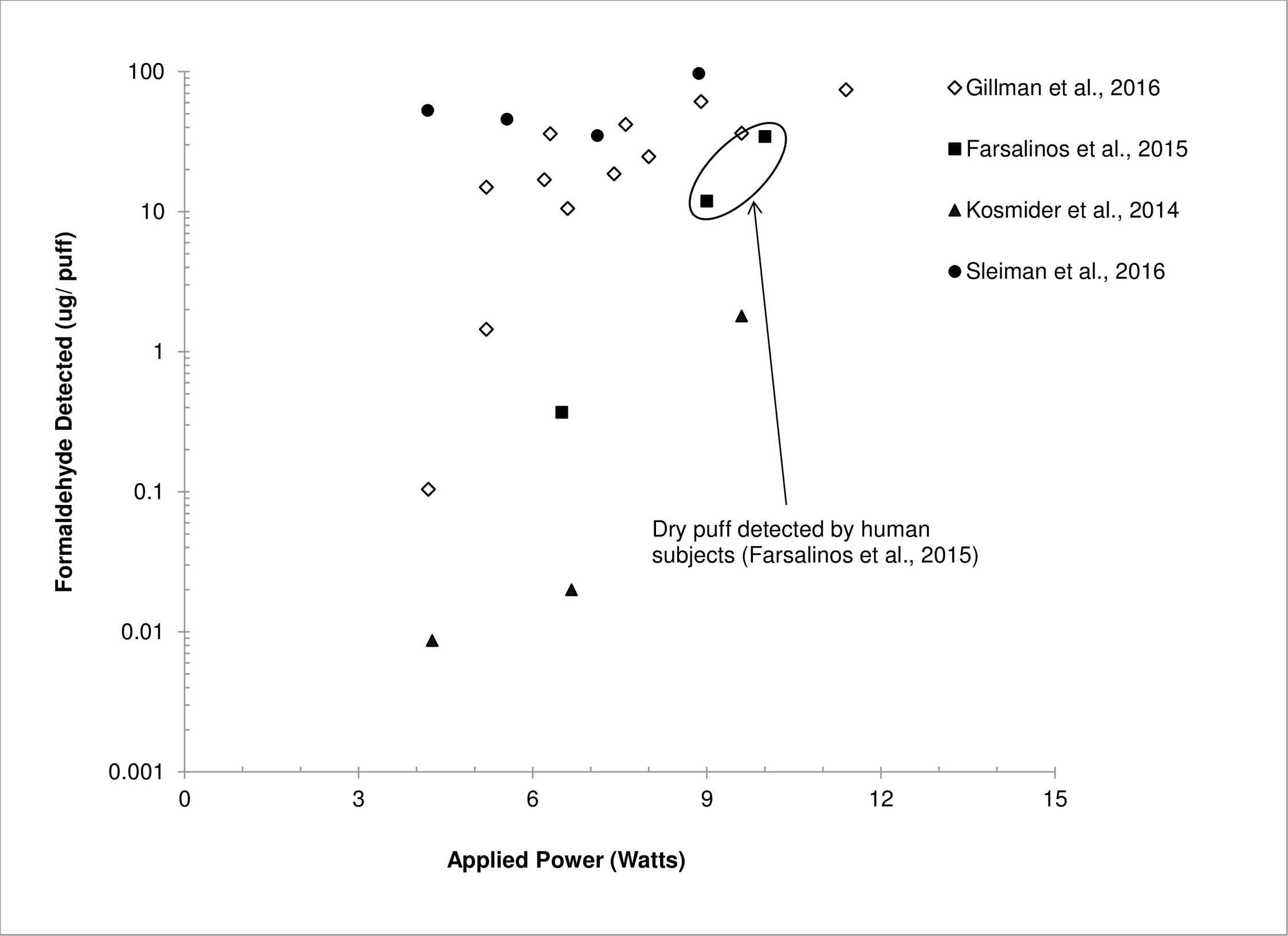

Supplement: S1 Fig — Note: a 1:1 mixture of propylene glycol and glycerol (PG/GL), with or without nicotine and water, was used for tests summarized here. (TIF) [file pone.0195925.s001.tif]
