# Supplementary material for: Measurement of heating coil temperature for e-cigarettes with a “top-coil” clearomizer
Source: PLoS One. 2018 Apr 19;13(4):e0195925. doi: 10.1371/journal.pone.0195925 (PMC5908153; doi:10.1371/journal.pone.0195925)
Supplement: S1 Table — (DOCX) [file pone.0195925.s002.docx]

**S1 Table: Details of e-cigarette settings and raw emission data for S1 Fig**

| **Reference** | **Coil #** | **Resistance (Ω)** | **Voltage (V)** | **Power (W)** | **Formaldehyde** | **Acetaldehyde** | **Acrolein** |
| --- | --- | --- | --- | --- | --- | --- | --- |
| Kosmider et al., 2014 ^8^ | EGO-3 | 2.4 | 3.2 | 4.3 | 0.13 µg/15 puffs | 0.43 µg/15 puffs | 0 µg/15 puffs |
|  | EGO-3 | 2.4 | 4 | 6.7 | 0.3 µg/15 puffs | 1.5 µg/15 puffs | 0 µg/15 puffs |
|  | EGO-3 | 2.4 | 4.8 | 9.6 | 27 µg/15 puffs | 1.73 µg/15 puffs | 0 µg/15 puffs |
| Farsalinos et al., 2015 ^10^ | A2 |  |  | 6.5 | 3.7 µg/10 puffs | 0.8 µg/10 puffs | 0.2 µg/10 puffs |
|  | A2 |  |  | 7.5 | 0 µg/10 puffs | 0.8 µg/10 puffs | 1.3 µg/10 puffs |
|  | A2 |  |  | 9 | 119.2 µg/10 puffs | 58.9 µg/10 puffs | 48.4 µg/10 puffs |
|  | A2 |  |  | 10 | 344.6 µg/10 puffs | 206.3 µg/10 puffs | 210.4 µg/10 puffs |
| Gillman et al., 2016 ^11^ | 1 -Device 1 | 2.2 | 3.8 | 6.6 | 155 µg/25 puffs | 137 µg/25 puffs | 0.55 µg/25 puffs |
|  | 1 -Device 1 | 2.2 | 3.8 | 6.6 | 372 µg/25 puffs | 253 µg/25 puffs | 11 µg/25 puffs |
|  | 2 -Device 1 | 2.8 | 3.8 | 5.2 | 174 µg/25 puffs | 144 µg/25 puffs | 3.6 µg/25 puffs |
|  | 2 -Device 1 | 2.8 | 3.8 | 5.2 | 575 µg/25 puffs | 497 µg/25 puffs | 12 µg/25 puffs |
|  | 3 -Device 1 | 3.4 | 3.8 | 4.2 | 3.3 µg/25 puffs | 1.7 µg/25 puffs | 0.55 µg/25 puffs |
|  | 3 -Device 1 | 3.4 | 3.8 | 4.2 | 1.9 µg/25 puffs | 1.5 µg/25 puffs | 0.55 µg/25 puffs |
|  | 1 -Device 1 | 2.2 | 4.2 | 8 | 754 µg/25 puffs | 491 µg/25 puffs | 32 µg/25 puffs |
|  | 1 -Device 1 | 2.2 | 4.2 | 8 | 485 µg/25 puffs | 360 µg/25 puffs | 15 µg/25 puffs |
|  | 2 -Device 1 | 2.8 | 4.2 | 6.3 | 893 µg/25 puffs | 800 µg/25 puffs | 0.55 µg/25 puffs |
|  | 2 -Device 1 | 2.8 | 4.2 | 6.3 | 907 µg/25 puffs | 808 µg/25 puffs | 22 µg/25 puffs |
|  | 3 -Device 1 | 3.4 | 4.2 | 5.2 | 22.9 µg/25 puffs | 8.5 µg/25 puffs | 0.55 µg/25 puffs |
|  | 3 -Device 1 | 3.4 | 4.2 | 5.2 | 49.3 µg/25 puffs | 27 µg/25 puffs | 0.55 µg/25 puffs |
|  | 1 -Device 1 | 2.2 | 4.6 | 9.6 | 858 µg/25 puffs | 628 µg/25 puffs | 25 µg/25 puffs |
|  | 1 -Device 1 | 2.2 | 4.6 | 9.6 | 958 µg/25 puffs | 663 µg/25 puffs | 40 µg/25 puffs |
|  | 2 -Device 1 | 2.8 | 4.6 | 7.6 | 976 µg/25 puffs | 850 µg/25 puffs | 26 µg/25 puffs |
|  | 2 -Device 1 | 2.8 | 4.6 | 7.6 | 1123 µg/25 puffs | 972 µg/25 puffs | 36 µg/25 puffs |
|  | 3 -Device 1 | 3.4 | 4.6 | 6.2 | 390 µg/25 puffs | 259 µg/25 puffs | 12 µg/25 puffs |
|  | 3 -Device 1 | 3.4 | 4.6 | 6.2 | 456 µg/25 puffs | 419 µg/25 puffs | 14 µg/25 puffs |
|  | 1 -Device 1 | 2.2 | 5 | 11.4 | 1240 µg/25 puffs | 771 µg/25 puffs | 53 µg/25 puffs |
|  | 1 -Device 1 | 2.2 | 5 | 11.4 | 2480 µg/25 puffs | 2036 µg/25 puffs | 593 µg/25 puffs |
|  | 2 -Device 1 | 2.8 | 5 | 8.9 | 1426 µg/25 puffs | 1181 µg/25 puffs | 54 µg/25 puffs |
|  | 2 -Device 1 | 2.8 | 5 | 8.9 | 1629 µg/25 puffs | 1264 µg/25 puffs | 84 µg/25 puffs |
|  | 3 -Device 1 | 3.4 | 5 | 7.4 | 639 µg/25 puffs | 641 µg/25 puffs | 31 µg/25 puffs |
|  | 3 -Device 1 | 3.4 | 5 | 7.4 | 292 µg/25 puffs | 214 µg/25 puffs | 12 µg/25 puffs |
| Sleiman et al., 2016 ^12^ | EGO-CE4 | 2.6 | 3.3 | 4.2 | 53 µg/puff | 10 µg/puff | 3 µg/puff |
|  | EGO-CE4 | 2.6 | 3.8 | 5.6 | 45.7 µg/puff | 9.2 µg/puff | 8.5 µg/puff |
|  | EGO-CE4 | 2.6 | 4.3 | 7.1 | 35 µg/puff | 31.8 µg/puff | 15.8 µg/puff |
|  | EGO-CE4 | 2.6 | 4.8 | 8.9 | 97 µg/puff | 50 µg/puff | 21.5 µg/puff |
