# Supplementary material for: Measurement of heating coil temperature for e-cigarettes with a “top-coil” clearomizer
Source: PLoS One. 2018 Apr 19;13(4):e0195925. doi: 10.1371/journal.pone.0195925 (PMC5908153; doi:10.1371/journal.pone.0195925)
Supplement: S2 Table — (DOCX) [file pone.0195925.s003.docx]

**S2 Table: Summary of temperature measurements at (or near) a heating coil reported in the literature**

| Reported Temperature | Measurement Method | E-cigarette Type | Test Condition | Reference |
| --- | --- | --- | --- | --- |
| ~120 – 950°C | infrared camera | 3^rd^ generation product with variable power setting | - mouthpiece removed - no puff flow - commercial e-liquid and its pure single constituents - dry coil tested (380 – 950°C) - liquid-drenched wick tested (~120 – 320°C) | 6 |
| 40 – 65°C | thermocouple | 1^st^ generation product | - simulated-use with puff flow - commercial e-liquid | 14 |
| >350°C | infrared camera | 2^nd^ generation product | - mouthpiece removed - no puff flow - dry coil | 15 |
| ~130 – ~340°C | infrared camera | e-cigarette with direct dripping atomizer | - mouthpiece removed - puff flow by reverse-puffing apparatus - commercial PG-based e-liquid - direct dripping on atomizer | 16 |
| 138 – 231°C | thermocouple | 1^st^ or 2^nd^ generation products | - simulated-use with puff flow - commercial e-liquids - various vaping topography (puff time and flow rate) | 17 |
| 116 – >217°C | infrared thermometer | 2^nd^ generation product | - mouthpiece removed - no puff flow - commercial e-liquid - dry coil tested (> 217°C) - full-wet coil tested (116°C) | 18 |
| ~275 – 475°C | infrared camera | prototype, no further specification | - mouthpiece removed - puff flow by reverse-puffing apparatus - commercial e-liquid | 19 |
